# Duration of surfactant administration and its impact on stabilisation of vital parameters in very preterm neonates <28 weeks of gestational age (SurfStab): 1 min versus 5 min—study protocol for a prospective randomised-controlled phase IV trial

**DOI:** 10.3389/fped.2026.1939771

**Published:** 2026-09-30

**Authors:** C. H. Wolfsberger, B. Schwaberger, N. Baik-Schneditz, A. Avian, M. Holter, G. Pichler

**Affiliations:** 1 Division of Neonatology, Department of Pediatrics and Adolescent Medicine, Medical University of Graz, Graz, Austria; 2 Research Unit for Neonatal Micro- and Macrocirculation, Medical University of Graz, Graz, Austria; 3 Institute of Medical Informatics, Statistics and Documentation, Medical University of Graz, Graz, Austria

**Keywords:** cerebral oxygen saturation, cerebral oxygenation, LISA, preterm neonates, surfactant, vital parameter, vital parameter stability

## Abstract

**Background:**

Respiratory distress syndrome remains one of the leading causes of morbidity and mortality in extremely preterm neonates. Surfactant replacement therapy substantially improves survival. Current European Consensus Guidelines recommend the less invasive surfactant administration (LISA) technique. However, no evidence exists on whether the duration of administration affects physiological stability. Rapid surfactant administration may provoke transient hypoxia or bradycardia due to airway obstruction, whereas slower delivery could potentially reduce such fluctuations.

**Methods/design:**

The SurfStab Trial is a single-centre, randomised, controlled phase IV clinical trial conducted at the Division of Neonatology, Medical University of Graz, Austria. Extremely preterm neonates (< 28 weeks of gestation) requiring surfactant via LISA will be randomly allocated (1:1) to receive surfactant administration over either 1 min (“1-min group”) or 5 min (“5-min group”). The primary outcome is the maximum change in cerebral oxygen saturation (crSO_2_), measured continuously using near-infrared spectroscopy (NIRS) from 5 min before LISA catheter insertion (baseline) to 15 min after completion of the procedure. Secondary outcomes include changes in arterial oxygen saturation (SpO_2_), heart rate (HR), arterial blood pressure (ABP), thoracic electrical impedance tomography (EIT), lung ultrasound, and the need for repeat surfactant administration, invasive ventilation, or supplemental oxygen up to three and up to 48 h after study intervention, as well as short-term outcomes at hospital discharge. The planned sample size is 76 neonates.

**Discussion:**

This trial will address a crucial gap in neonatal care by determining whether the duration of surfactant administration influences cerebral and systemic oxygen stability in extremely preterm neonates. The findings may refine evidence-based recommendations for optimal LISA procedure and may contribute to improved outcomes.

**Clinical Trial Registration:**

ClinicalTrials.gov, Identifier: NCT NCT07261787. Registered 02/12/2025. Eu CT number 2025-522754-39-00. Registered 01/12/2025.

## Background

### Prematurity and respiratory distress syndrome (RDS)

On a global scale, approximately 11% of neonates are delivered before completing 37 weeks of gestation, resulting in an estimated annual incidence of 13–15 million premature births. In Austria, 6.8% of infants were born prematurely in 2024, with survival rates continuing to improve due to advances in neonatal intensive care. Extreme prematurity, defined as birth before completed 28 weeks of gestation, remains associated with high morbidity and mortality, including bronchopulmonary dysplasia (BPD), intraventricular haemorrhage (IVH), or neurodevelopmental impairment (1–3).

Respiratory distress syndrome (RDS), a hyaline membrane disease caused by surfactant deficiency, is the predominant respiratory morbidity during the early neonatal period in these neonates (4, 5). The incidence of RDS decreases with increasing gestational age (6). Among preterm neonates born at 23–28 weeks of gestation, the incidence of RDS is nearly 100%, particularly among those born before 26 weeks of gestational age (4, 6). Several factors increase the risk of RDS, including male sex, elective delivery without labour, perinatal hypoxia, and maternal diabetes during pregnancy (7, 8). The pathophysiological mechanism of RDS is impaired or delayed production and secretion of surfactant. Clinical manifestations include dyspnoea, grunting, retractions, cyanosis, and respiratory failure (9, 10). Furthermore, complications such as pneumothorax, pulmonary haemorrhage, persistent pulmonary hypertension in newborns, and subsequent BPD are associated with RDS in preterm neonates (11, 12).

### Surfactant replacement therapy

Since the introduction of surfactant replacement therapy for RDS treatment in the early 1990s, as first described by Jobe et al., morbidity and mortality of preterm neonates have significantly decreased (13). According to the 2022 European Consensus Guidelines on the Management of RDS, natural surfactant delivered via the “less invasive surfactant administration” (LISA) technique should be preferred. Surfactant should be administered when the fraction of inspired oxygen (FiO_2_) exceeds 0.3 while the neonate is on continuous positive airway pressure (CPAP) at ≥6 cm H_2_O (14). In recent years, lung ultrasound has emerged as an important bedside tool to support the diagnosis of RDS and to guide the decision for surfactant administration. Several studies demonstrated that lung ultrasound scores predict surfactant requirement more accurately than FiO_2_ thresholds alone and may allow earlier and more individualised treatment. Nevertheless, FiO_2_ thresholds according to the European Consensus Guidelines remain the standard indication in many centres (14–16). An initial dose of 200 mg/kg poractant alfa is recommended, with repeat doses of surfactant (second or third) considered in cases of persistent RDS.

Several techniques for surfactant administration have been described, including LISA, Intubation-Surfactant-Extubation (INSURE), surfactant administration via a laryngeal mask or supraglottic airway (SALSA) and nebulised surfactant administration. Among the less invasive approaches, LISA and SALSA are increasingly used, whereby LISA is currently considered the preferred less invasive approach for spontaneously breathing preterm neonates.

During LISA, a thin catheter is placed into the trachea while the neonate continues to breathe spontaneously and receives non-invasive respiratory support (17, 18). The catheter should be inserted 1.5–2.0 cm below the vocal cords under direct or video laryngoscopy while the neonate continues to breathe spontaneously and receives non-invasive respiratory support. Surfactant should then be instilled over 2–5 min at a rate of 0.5–1.0 ml/min. A further study described a recommended duration of 1–2 min, administering surfactant in four aliquots followed by 1 ml of air (19). Administration of surfactant is associated with a number of acute side effects, including failure to insert the catheter, significant surfactant reflux, hypoxia, bradycardia, or the need for positive pressure ventilation during LISA (20). To date, no consensus exists regarding the optimal duration of surfactant administration during the LISA procedure.

### Cerebral oxygenation and surfactant administration

Cerebral oxygen saturation (crSO_2_), measured using near-infrared spectroscopy (NIRS), reflects the balance between cerebral oxygen delivery and consumption by providing continuous, non-invasive monitoring. Several studies have examined crSO_2_ in the context of surfactant administration, summarised in a systematic review (21). Some studies focused on the effect of surfactant administration on crSO_2_ (22–30), whereas others compared different methods of administration (31–36). Although the methodology, outcome measures, and NIRS devices used varied between studies, the overall finding was a decrease in crSO_2_ during and immediately after surfactant administration, followed by a return to baseline levels shortly thereafter. However, most studies investigated crSO_2_ in the context of surfactant administration in intubated neonates (22–26, 30, 33–35, 37).

The impact of surfactant administration using the LISA technique on crSO_2_ has been described by Hanke et al., Bertini et al., and Li et al. (31, 32, 36). Bertini et al. observed a significant decrease in crSO_2_ during the LISA procedure to 45.5 ± 10.6% from a baseline of 75.6 ± 7.9% (32). Sixty minutes after the surfactant administration, the crSO_2_ increased to 79.7 ± 7.3%, exceeding baseline levels. Cerebral fractional tissue oxygen extraction (cFTOE) showed an opposite pattern to crSO_2_, with a significant increase during surfactant administration. Similarly, Li et al. described a decrease in crSO_2_ during the LISA procedure, with a 5.9% drop from baseline. Within the first ten minutes after surfactant administration, crSO_2_ almost returned to baseline values again (36). Hanke et al. described no significant differences in crSO_2_ between extremely and very preterm neonates who only received nasal CPAP and those undergoing the LISA procedure (31); nevertheless, preterm neonates in the LISA group showed a non-significant decrease in crSO_2_ of approximately 20% from baseline during this procedure. Overall, surfactant administration via the LISA technique is associated with a transient reduction in crSO_2_ followed by a recovery to baseline values shortly after the procedure.

### Rationale for the SurfStab trial

In the absence of evidence-based guidelines on the optimal duration of surfactant administration, the SurfStab Trial aims to compare physiological stability between two administration durations – 1 min versus 5 min – both of which fall within current guideline recommendations. By using continuous crSO_2_ monitoring, this trial seeks to determine whether slower administration improves stability during and after surfactant administration in extremely preterm neonates.

Accordingly, the SurfStab trial will evaluate whether the duration of surfactant administration (“1-min” vs. “5-min”) using LISA technique affects the primary outcome measure, change in crSO_2_, as well as secondary outcome measures, including arterial oxygen saturation (SpO_2_) and heart rate (HR), during the LISA procedure and within the first three hours thereafter in preterm neonates born at <28 weeks of gestation.

## Methods

### Design

This investigator-initiated, prospective, single-centre randomised, controlled phase IV clinical trial is designed to compare two pre-specified durations of surfactant administration during the LISA procedure in extremely preterm neonates. The trial will be conducted exclusively at the Division of Neonatology, Department of Paediatrics and Adolescent Medicine, Medical University of Graz, Austria. A total of 76 preterm neonates (38 per group) with a gestational age <28 weeks will be enrolled (Figure 1). The trial follows the SPIRIT guidelines for clinical trial protocols (38) and complies with the EU Clinical Trials Regulation (EU-No. 536/2024), ICH-GCP principles, and the Declaration of Helsinki (39). The trial was registered at clinicaltrials.gov (NCT07261787).

**Figure 1:**
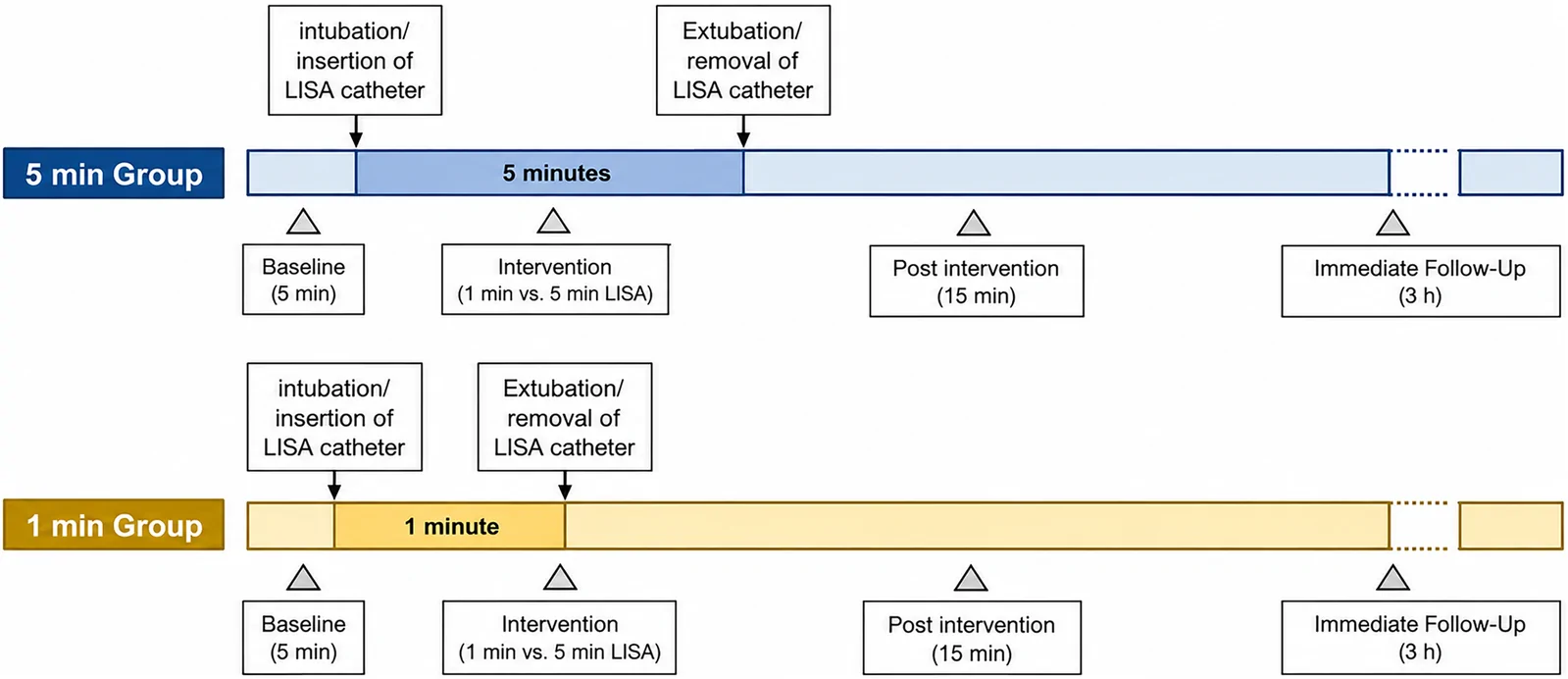
Trial flow chart.

### Study population

Preterm neonates born at <28 weeks of gestation are eligible for inclusion.

Additional inclusion criteria are a clinical decision to perform surfactant administration via the LISA procedure according to routine clinical practice, and a postnatal age of <72 h at the time of enrolment. The indication for surfactant administration is not determined by the study protocol. Exclusion criteria include preterm neonates requiring invasive ventilation or receiving surfactant via the INSURE procedure, severe pulmonary or cardiac malformations affecting oxygenation, congenital cerebral malformation, and pre-existing diagnosis of IVH > grade 2 or periventricular haemorrhage (PVH), if identified as part of routine clinical care. Cranial ultrasound is not required by the study protocol prior to surfactant administration.

### Sample size and statistical analyses

The planned sample size of 76 neonates was derived from anticipated differences in the primary endpoint. The sample size calculation was based on prior studies assessing crSO₂ during LISA procedures (21, 32, 36), which reported a decrease from 78.7% ± 9.1 to approximately 61.1% ± 11.5. A 10% decrease in crSO₂ is considered clinically relevant. To detect a difference of 7.5% (about 10% decrease from the original value) in means between groups, with a common standard deviation of 11.5, a sample size of 38 participants per group is required. This provides a statistical power of 80% (1-*β* = 0.80) to detect a clinically relevant difference using a two-sided significance level of 5% (*α* = 0.05). The effect size corresponds to *δ* = 0.65.

The primary analysis will follow the intention-to-treat principle, including all randomised neonates in the groups to which they were allocated. To evaluate the primary hypothesis – whether the duration of surfactant administration during the LISA procedure results in a difference in the maximum change in crSO_2_ from baseline to 15 min after completion of the LISA procedure (defined as removal of the thin LISA catheter) – a linear regression model will be applied. The dependent variable will be the maximum change in crSO_2_, the independent variables will include treatment group (“1-min group” vs. “5-min group”) and the stratification factor used in randomisation (gestational age: <26 + 0 weeks vs. ≥26 + 0 to <28 + 0 weeks). Maximum change will be calculated as the difference between the baseline value and crSO_2_ value within the primary time window. Baseline is defined as the mean crSO_2_ value during the five minutes before catheter insertion. CrSO_2_ will be documented minutely starting five minutes before the procedure (= baseline) until 15 min after the LISA catheter was removed (Figure 2).

**Figure 2:**
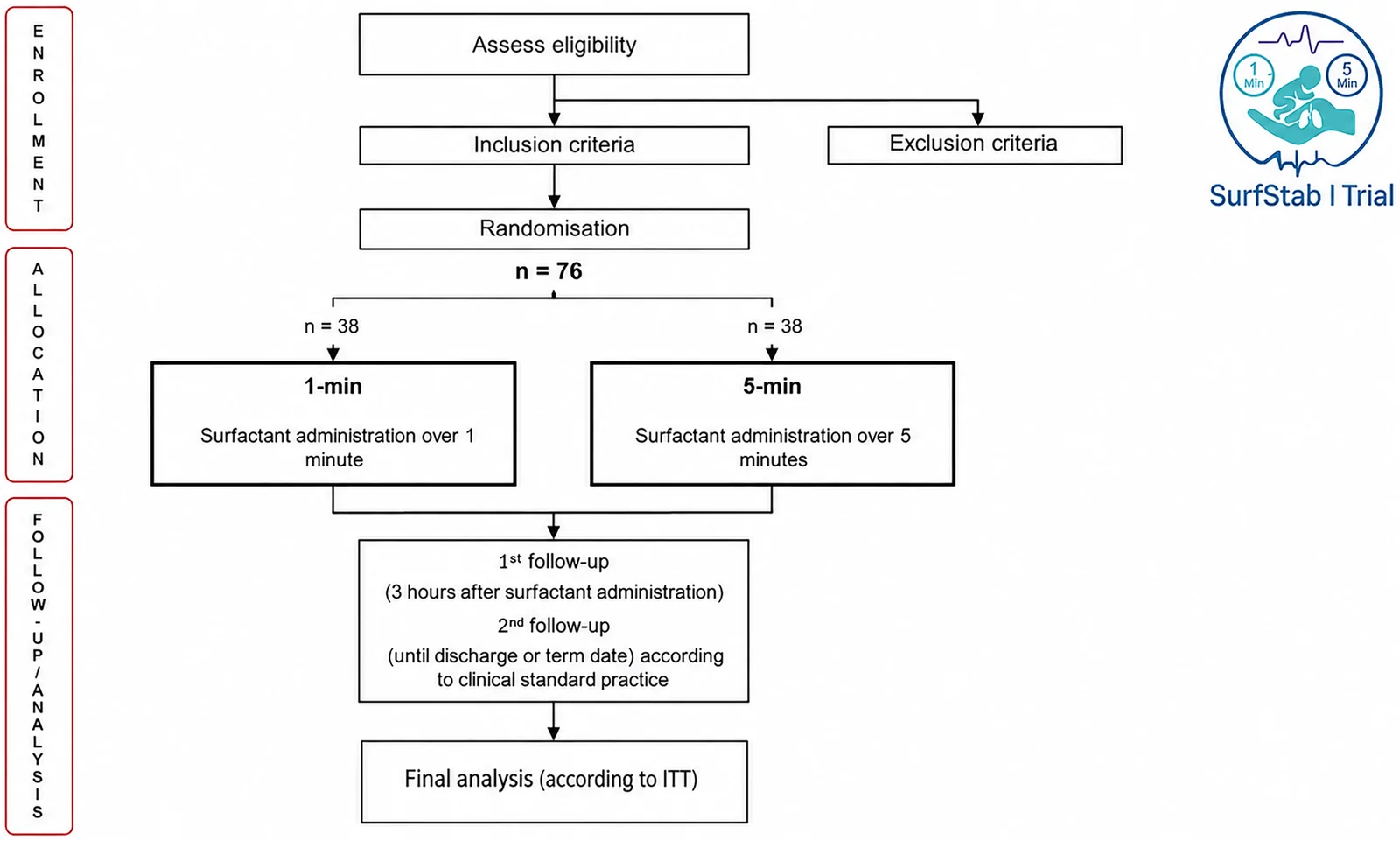
Assessment of outcome. LISA, less invasive surfactant administration.

For all secondary endpoints, a two-sided significance level of 5% will be applied. Group differences between the 1-min group and 5-min group in maximum changes of SpO₂ and HR from baseline to 15 min after removal of the LISA catheter will be analysed using independent *t*-tests or Mann–Whitney *U*-tests, as appropriate. Maximum change will be defined as the difference between baseline values and the maximum or minimum recorded value within the predefined time window.

Further secondary analyses will assess group differences in maximum changes of SpO₂, HR, crSO₂, and mean arterial blood pressure (MABP) from baseline up to three hours after surfactant administration, as well as supplemental oxygen requirement and the cumulative duration of bradycardia and hypoxia during this period, using *t*-tests or Mann–Whitney *U*-tests. Longitudinal courses of SpO₂, HR, crSO₂, MABP, and FiO₂ during the first three hours after the LISA procedure will be analysed using linear mixed-effects models including time and group as fixed effects.

Binary secondary outcomes, including the need for repeated surfactant administration, invasive ventilation within two days after surfactant administration, and short-term outcomes (cerebral injury, neonatal morbidities, and mortality until discharge or term-equivalent age), will be compared between groups using chi-square or Fisher's exact tests, and relative risks with 95% confidence intervals will be reported.

Exploratory analyses will include descriptive comparisons of demographic characteristics, prenatal and perinatal history, and early postnatal transition variables between groups. Data related to the LISA procedure, including postnatal age at treatment, surfactant dose, catheter type, and number of catheterisation attempts, will be analysed using *t*-tests or Mann–Whitney *U*-tests for continuous variables and chi-square or Fisher's exact tests for categorical variables. Respiratory support, medication use, and the need for mechanical ventilation during the first two days and the first week after the LISA procedure will be compared using chi-square or Fisher's exact tests.

Blood gas analyses obtained after birth, before and after the LISA procedure, and within three hours after surfactant administration, as well as transcutaneous CO₂ monitoring data during the first three hours, will be compared between groups using *t*-tests or Mann–Whitney *U*-tests. Lung ultrasound scores, assessed before, three hours and two days after the LISA procedure, will be analysed using parametric or non-parametric tests, as appropriate. To assess differences in lung ventilation and lung recruitment, using electrical impedance tomography, starting before LISA procedure and lasting for 72 h, maximum changes from baseline will be calculated and compared between groups using *t*-tests or Mann–Whitney *U*-tests. Neuromonitoring outcomes, including amplitude-integrated electroencephalography (aEEG) during the first week after the LISA procedure, neuroimaging [magnetic resonance imaging (MRI) and/or cerebral ultrasound], and general movement assessments until discharge or term-equivalent age, will be compared using chi-square or Fisher's exact tests.

### Randomisation

Randomisation will be performed via the web-based REDCap® randomisation module. After verifying eligibility, the investigator enters the neonate's gestational age and date of birth into the REDCap system, which then automatically assigns the subject to either the “1-min” or “5-min” group according to a pre-generated randomisation list. The list is stored securely within REDCap, with access restricted to the data manager. The allocation result is displayed immediately after entry and automatically documented within the electronic Case Report Form (eCRF), ensuring concealed allocation.

Randomisation will be stratified by gestational age (<26 + 0 weeks vs. ≥26 + 0 to <28 + 0 weeks) to ensure comparable distributions in both treatment groups (“1-min” and “5-min”), as gestational age is a relevant prognostic factor influencing cerebral oxygenation and response to surfactant therapy. The allocation ratio within each stratum will be 1:1.

### Deferred consent procedure

Recruitment will take place in the neonatal intensive care unit, where neonates eligible for LISA are managed. Given the acute nature of RDS in extremely preterm neonates and the urgent need for surfactant administration, the study will use a deferred consent procedure. Deferred consent will be obtained after the enrolment and randomisation of the neonate. Therefore, the allocated intervention (surfactant over either 1 or 5 min) will be performed before written parental consent is obtained. This approach is ethically justified, as it prevents selection bias and ensures that both planned and emergency LISA procedures can be represented, particularly in critically ill neonates for whom delaying treatment to obtain prospective parental consent may pose a clinical risk. Parents will be informed as soon as reasonably possible, and written consent will be sought in an undisturbed setting, with sufficient opportunity to ask questions. Parents retain the right to withdraw consent at any time, even after the intervention. As both intervention durations are within current guideline recommendations and routine clinical practice, no participant is exposed to treatment outside the accepted standard of care. The trial adheres to ethical principles outlined in the Declaration of Helsinki and ICH-GCP guidelines and has been approved by the relevant ethics committee and regulatory authorities.

### Duration of interventions

The intervention consists of surfactant administration over either one minute (“1-min group”) or five minutes (“5-min group”), according to randomisation. In addition to routine monitoring, crSO_2_ monitoring will begin five minutes before surfactant administration and will continue for three hours thereafter. Thus, the intervention period corresponds to the randomised administration duration (1 min vs. 5 min), while the monitoring period will comprise the five-minute baseline phase before the surfactant administration, the intervention itself, and the three hours observational period following the LISA procedure (Figure 2).

### Duration of follow-up

The immediate follow-up will extend to three hours post-intervention (surfactant administration). Continuous monitoring of crSO_2_, SpO₂, HR, and other clinical parameters will be performed to assess early physiological responses and potential adverse events. The short-term follow-up will occur on day 2 and day 7 post-intervention and will include assessment of respiratory support requirements, evaluation of lung ventilation and recruitment using electrical impedance tomography (EIT), determination of need for repeat surfactant administration or invasive ventilation, lung and cerebral ultrasound examinations, and aEEG. The final follow-up will take place at term-equivalent age (37–42 weeks of gestational age) or at discharge, with assessment of short-term morbidities and optional cerebral imaging, including MRI, aEEG and general movement assessment.

### Monitoring

The antepartum medical history and birth-related data will be collected. Gestational age, birth weight, sex, umbilical artery pH and Apgar scores will be documented for each neonate. Monitoring will be performed according to routine clinical practice, including pulse oximetry for continuous SpO_2_ and pulse-derived HR monitoring. HR will be corroborated with ECG-derived HR for quality control. Non-invasive arterial blood pressure (ABP) will be measured using standard neonatal cuffs, while invasive ABP monitoring will be used when clinically indicated. CrSO_2_ will be measured using the INVOS 7100 Cerebral/Somatic Oximeter monitor (Medtronic, USA) with a neonatal sensor placed on the left forehead. This device uses a continuous-wave spatially resolved NIRS technique to measure crSO_2_ non-invasively. NIRS measurements will begin at least five minutes before the LISA procedure (baseline) and will continue for three hours thereafter. CrSO_2_, SpO_2_, HR, ABP, and FiO_2_ will be collected and documented every minute starting five minutes before the insertion of the LISA catheter, during the procedure, and up to 15 min thereafter. Monitoring data (crSO_2_, SpO_2_, HR, and ABP) will then be collected every five minutes from 15 min to three hours after the LISA procedure (Table 1).

**Table 1:** Schedule of enrolment, intervention and assessments.

Description	Before LISA procedure	5 min before LISA procedure	During LISA procedure	15 min after LISA procedure/stabilisation of the infant	3 h after LISA procedure	2 days after LISA procedure	7 days after LISA procedure	Term-equivalent age (37 + 0–42 + 0 weeks)/before discharge
Screening: In-/Exclusion criteria^a^	X							
Parental deferred consent^a^				X				
Randomisation^a^	X							
Antepartum medical history	X							
NIRS monitoring^a^		X	X	X	X			
Vital parameter monitoring		X	X	X	X			
Intervention^a^			X					
Adverse reaction^a^			X					
Assessment of secondary outcome (repetitive surfactant, invasive ventilation)^a^						X	X	
Lung ultrasound	X			(X)	X	X		
EIT	X	X	X	X	X	X		
Cerebral ultrasound						X	X	X
MRI (optional)								X
General movement assessments (optional)								X
aEEG (optional)						X	X	X
Neonatal morbidities								X

aEEG, amplitude-integrated electroencephalography; EIT, electrical impedance tomography, LISA, less invasive surfactant administration; MRI, magnetic resonance imaging; NIRS, near-infrared spectroscopy.

a Study specific interventions.

### Interventions

The decision to perform a LISA procedure for surfactant replacement therapy will be made by the attending clinician according to routine clinical practice and existing guidelines (31) and will not be influenced by participation in the trial. The LISA procedure will be performed according to routine using poractant alfa (Curosurf, Chiesi, Italy), a porcine lung extract. The dosage used will follow standard practice and will not be influenced by the trial. The type of LISA catheter used will be determined according to local routine clinical practice and at the discretion of the attending clinician. The choice of catheter will not be influenced by the trial protocol. The duration of laryngoscopy and the level of experience of the clinician performing the LISA procedure (e.g., resident or consultant neonatologist) will be documented for each procedure. According to randomisation, the duration of surfactant administration will differ between the two groups. Once the decision to perform LISA procedure has been made, the randomisation will be carried out. CrSO_2_ monitoring will begin at least five minutes before insertion of the LISA catheter.

### Groups

#### 1-min group

The LISA procedure will be performed according to local clinical practice, except for the duration of surfactant administration, which will be 1 min in this randomisation arm. The amount of surfactant will be chosen at discretion of the attending clinician and according to standard practice.

#### 5-min group

The LISA procedure will be performed according to local clinical practice, except for the duration of surfactant administration, which will be 5 min in this randomisation arm. The amount of surfactant will be chosen at discretion of the attending clinician according to standard practice.

### Outcome measure

The primary outcome measure will be the maximum change in crSO_2_ from baseline to the end of the primary observation window, defined as the duration of surfactant administration (1 or 5 min according to randomisation) plus 15 min after LISA catheter removal. CrSO_2_ will be recorded every minute from five minutes before surfactant administration until 15 min after LISA catheter removal (Figure 2). Baseline will be defined as the mean crSO_2_ value during the five minutes before the start of the intervention.

The secondary outcome measures will include the maximum change in SpO_2_ and HR from baseline to the end of the primary observation window. These parameters will be recorded every minute from five minutes before surfactant administration until 15 min after LISA catheter removal. Baseline will be defined as the mean SpO_2_ and HR values during the five minutes before the start of the intervention.

Afterwards, changes in crSO_2_, SpO_2_, HR, and ABP will be collected every five minutes from 15 min to three hours after the LISA procedure.

Additional secondary outcome measures will include the need for supplemental oxygen, total duration of bradycardia and hypoxia (in minutes) up to three hours after surfactant administration, the need for repeated surfactant administration, the requirement of invasive ventilation during the first two days after initial surfactant administration and short-term outcome until hospital discharge. The latter include IVH (any grade), PVH, periventricular leukomalacia (any grade), BPD, retinopathy of prematurity, necrotizing enterocolitis, persistent ductus arteriosus (requiring therapy) and sepsis.

Exploratory outcome measures will include respiratory and medical support before and during the first week after the LISA procedure, blood gas analyses (including pH, partial pressure of carbon dioxide, partial pressure of oxygen, base excess, glucose concentration, and lactate concentration) before and within three hours after the LISA procedure, transcutaneous CO_2_ before and during the three hours after the LISA procedure, lung ventilation and recruitment assessed by EIT starting five minutes before LISA procedure, lasting for 72 h, aEEG during the first week after the LISA procedure and before discharge, cerebral imaging (MRI or cranial ultrasound) until term-equivalent age, and general movement assessment until hospital discharge. Further exploratory outcome measures will include lung ultrasound assessed using the lung ultrasound score according to Rodriguez-Fanjul et al., performed before the LISA procedure, within the first three hours after the LISA procedure, and two days after the LISA procedure (40). Changes in lung ultrasound scores over time will be compared between the two intervention groups. Both, lung ultrasound and EIT are exploratory study assessments and will only be performed if they can be integrated into routine clinical care without delaying the clinically indicated surfactant administration. These assessments are not prerequisites for the LISA procedure and may be omitted if immediate surfactant administration is required.

Exploratory outcome measures will also include procedure-related complications, including severe or prolonged bradycardia (HR < 80 bpm for >60 s), prolonged desaturation (SpO_2_ < 80% for >60 s), surfactant reflux during the LISA procedure, the need for positive-pressure ventilation (PPV) during the LISA procedure, failed catheterisation, and the need for immediate intubation after the LISA procedure.

### Blinding

The trial is randomised but unblinded, as blinding is not feasible due to the visible nature of the intervention duration. However, the use of objective monitoring and standardised data collection procedures will minimise the risk of observer bias.

### Data management

Data collection and management will be carried out using REDCap, a GCP-compliant eCRF system widely used in academic clinical research. REDCap ensures traceability, data integrity, and secure, role-based access for all authorized trial personnel. Built-in validation rules check data for completeness, plausibility, and consistency at the time of entry. In addition, trial monitors may perform further manual data checks during on-site or remote monitoring visits. Any discrepancies identified will be addressed through the query management function in REDCap. All queries, responses, and data modifications are fully documented in the audit trail to ensure transparency and traceability. Monitoring visits will be conducted regularly to verify source data, check for protocol compliance, and ensure that data entry procedures meet GCP and institutional quality standards. The database will be locked after all protocol-relevant data have been entered and verified and all queries have been resolved.

### Safety

The preterm patient population is highly vulnerable and often critically ill. Most adverse events may be serious in nature, irrespective of participation in the SurfStab trial and exposure to the intervention. Therefore, a high proportion of serious adverse events (SAEs) is expected in both groups. All SAEs occurring during the study period will be recorded. Given the high incidence of serious clinical events related to prematurity and the underlying condition of the study population, not all SAEs can be meaningfully attributed to the study intervention. Mortality will be recorded and reported as an SAE irrespective of its relationship to the study participation. In addition, all adverse events occurring in temporal association with the LISA procedure or surfactant administration will be systematically documented in this trial, regardless of their seriousness or relationship to the study participation. Procedure-related adverse events include severe or prolonged bradycardia (HR < 80 bpm for >60 s), prolonged desaturation (SpO_2_ < 80% for >60 s), surfactant reflux during LISA procedure, need for PPV during the LISA procedure and failed catheterisation, or the need for intubation immediately after the LISA procedure. These procedure-related adverse events will serve as predefined stopping criteria for the study intervention. If any of these events occur, the LISA procedure will be discontinued and further management will be at the discretion of the responsible physician according to local clinical practice, without any study-mandated requirements regarding continuation or modification of the LISA procedure.

Safety monitoring will be performed once 20% of the planned sample size has been enroled, in order to assess SAEs judged as having a “certain” or “probable/likely” relationship to the duration of surfactant administration via the LISA procedure (“1-min” vs. “5-min” groups) or to the cerebral NIRS sensor. All enroled preterm neonates will be insured in accordance with applicable legislation.

### Ethical consideration

The SurfStab trial will initiate participant randomisation only after approvals from the relevant ethics committee and regulatory authorities have been obtained via the Clinical Trials Information System (CTIS), and after all study staff have completed trial-specific training. The participants in this study are preterm neonates (<28 weeks of gestation), a population incapable of providing informed consent. Therefore, informed consent is obtained from the parents or legal guardians.

Given the acute nature of RDS in extremely preterm neonates and the need for timely surfactant administration, a deferred consent procedure will be used. Deferred consent will be obtained as soon as reasonably possible after the inclusion and randomisation of the neonate. The parents retain the right to withdraw consent at any time, even after the intervention.

## Discussion

The SurfStab trial addresses a specific clinical question regarding the optimal duration of surfactant administration via the LISA procedure in extremely preterm neonates – a topic for which evidence is limited and partly conflicting, particularly concerning its impact on cerebral oxygenation. While surfactant treatment is unequivocally beneficial and recommended by consensus guidelines (14), procedural variability exists, particularly with respect to the duration of surfactant administration during LISA. This study aims to provide critical insights into whether different administration durations – specifically 1 versus 5 min – affect vital parameters, primarily crSO₂, as well as SpO₂ and HR. This investigation seeks to determine whether a longer duration of surfactant administration during the LISA procedure is associated with better physiological stability and improved short-term clinical outcomes in this vulnerable population.

The primary importance of this investigation lies in the particular vulnerability of the preterm brain to fluctuations in oxygen delivery. Even transient episodes of cerebral desaturation or haemodynamic instability may contribute to white matter injury and subsequent neurodevelopmental impairment (41). By focusing on crSO₂ as the primary outcome and linking it to short-term outcomes, the trial will provide initial evidence on whether modifying clinical practice, specifically by slowing the duration of surfactant administration, can reduce cerebral hypoxic burden and thereby improve physiological stability and potentially neurodevelopmental outcomes in this high-risk population.

A systematic review investigating cerebral oxygenation in preterm neonates following surfactant administration revealed a consistent pattern: regardless of the method used, most studies reported a transient decrease in crSO₂, followed by a subsequent return to baseline levels (21). This initial decrease may be attributed to factors such as transient airway obstruction during bolus administration or an increase in cerebral blood flow resulting from augmented left ventricular output. Notably, one study using aerosolised surfactant administered via a jet nebulizer reported contrasting findings, observing an increase in crSO₂ after administration (29). This suggests that both the method and rate of surfactant delivery may significantly influence cerebral oxygenation.

Several plausible mechanisms may explain how the duration of surfactant administration influences cerebral oxygenation and cardiorespiratory stability. Rapid bolus instillation of surfactant may produce abrupt changes in surface tension and dynamic lung mechanics, temporarily impeding effective ventilation and increasing intrapulmonary shunt (42–44). Conversely, more gradual administration may mitigate these rapid physiological shifts, potentially resulting in fewer pronounced fluctuations in cerebral oxygenation and improved haemodynamic stability. Faster instillation may also provoke reflex apnoea or laryngospasm, leading to brief hypoventilation and subsequent desaturation, whereas slower administration may be better tolerated by spontaneously breathing preterm neonates (45). Additionally, the volume of surfactant introduced rapidly may transiently increase intrathoracic pressure, impeding cerebral venous return and thus reducing cerebral oxygenation (42). Finally, rapid bolus delivery may increase the risk of surfactant reflux, reducing the proportion of dose reaching distal airways and potentially requiring repeat dosing.

### Strengths and limitations

The randomised design minimises selection bias, and both durations of surfactant administration align within current guidelines. Deferred consent preserves clinical equipoise and prevents the exclusion of the highest-risk preterm neonates who might benefit the most. A further strength is the homogeneity of the study population, as including only extremely preterm neonates (<28 weeks of gestation) reduces biological variability and increases the sensitivity to detect physiological effects. Although the procedural nature of the intervention precludes blinding of operators, the stratified randomisation helps to balance gestational age–related risk between groups. Nevertheless, residual differences in baseline respiratory drive, antenatal steroid exposure, or early comorbidities may influence tolerance to the LISA procedure and the crSO₂ response. In addition, the single-centre nature and operator-dependent variability may limit generalisability.

In conclusion, the SurfStab trial will rigorously evaluate whether the duration of surfactant administration during the LISA procedure affects cerebral and systemic stability in extremely preterm neonates, with the potential to inform current clinical practice and future consensus guidelines on surfactant therapy. If the trial identifies a clinically relevant advantage of one duration, multi-centre confirmatory trials will be warranted.

## Trial status

Protocol version 4.0; dated 4 December 2025. Recruitment began 26 January 2026 and is expected to be completed by August 2028.
